# Mental health in children and adolescents with overweight or obesity

**DOI:** 10.1186/s12889-023-15032-z

**Published:** 2023-01-19

**Authors:** Lucas-Johann Förster, Mandy Vogel, Robert Stein, Anja Hilbert, Julius Lars Breinker, Marleen Böttcher, Wieland Kiess, Tanja Poulain

**Affiliations:** 1 LIFE Leipzig Research Center for Civilization Diseases, Leipzig University, Philipp-Rosenthal-Strasse 27, 04103 Leipzig, Germany; 2 Department of Women and Child Health, University Hospital for Children and Adolescents and Center for Pediatric Research, Leipzig University, Liebigstrasse 20a, 04103 Leipzig, Germany; 3 Helmholtz Institute for Metabolic, Obesity and Vascular Research (HI-MAG) of the Helmholtz Zentrum München at the University of Leipzig and University Hospital Leipzig, Leipzig, Germany; 4 Department of Psychosomatic Medicine and Psychotherapy, Behavioral Medicine Research Unit, Integrated Research and Treatment Center Adiposity Diseases, University of Leipzig Medical Center, Stephanstrasse 9a, 04103 Leipzig, Germany

**Keywords:** BMI-SDS, Childhood, Health-related quality of life, Somatoform complaints, Behavioral strengths and difficulties

## Abstract

**Background:**

Overweight and obesity represent huge concerns for children's physical and mental well-being. This study examined the relationship between body mass index (BMI) and health-related quality of life (HRQoL), somatoform complaints, and behavioral problems in children and adolescents. Additionally, the influence of sex, age, and socioeconomic status (SES) on these associations was considered.

**Methods:**

In total, we studied 2350 participants between the ages of 4 and 18 years (1213 4- to 10-years-old (child sample) and 1137 11-to 18-year-olds (adolescent sample)). To assess HRQoL, somatoform complaints, and behavioral difficulties, we applied the KIDSCREEN-27, a short form of the Giessen Complaints Questionnaire, and the Strengths and Difficulties Questionnaire (SDQ). The BMI was transformed to BMI standard deviation scores (BMI-SDS), according to German gender- and age-specific reference data. Associations were investigated using linear regression analyses. Each association was checked for interaction with sex, age, and SES.

**Results:**

Regarding HRQoL, we found worsening scores in physical well-being and psychological well-being with increasing BMI-SDS. Somatoform complaints were not significantly associated with BMI-SDS. Conduct problems, peer relationship problems, and emotional problems (the latter only in the adolescent sample) were positively associated with BMI-SDS. While we did not observe any significant interactions with sex, we found some significant interactions with age and/or SES.

**Conclusion:**

Our findings highlight the importance of mental difficulties in children and adolescents with higher BMI and, consequently, underline the relevance of including psychological interventions in the treatment of overweight or obesity.

## Background

Obesity is a major health concern, not only in adults but also in children and adolescents, which implies many negative somatic and psychological consequences and risks [1–4]. According to the World Health Organization, the prevalence of obesity has almost tripled worldwide since 1975 [5]. In the "German Health Interview and Examination Survey for Children and Adolescents" (KiGGS) conducted from 2014 to 2017, the overweight (including obesity) prevalence in children and adolescents between 3 and 17 years was 15.4% and the obesity prevalence amounted to 5.9% [6]. In comparison with studies from the 1980s and 1990s, there has been a 50% rise in the overweight prevalence among children and adolescents, and the proportion of children and adolescents with obesity has over doubled [7]. The COVID-19 pandemic has further augmented this trend [8].

According to the KiGGS study, the prevalence of obesity and overweight does not differ between girls and boys [6]. However, the prevalence of both overweight and obesity tended to rise with age [6]. Moreover, the study suggested a significantly higher prevalence of overweight among children and adolescents of families with a low socioeconomic status (SES) in contrast to families with high and middle SES [6]. In boys, obesity was also significantly more prevalent in children and adolescents with low than high SES [6]. Interestingly, a recent review reported a negative association between obesity and SES in high-income countries but a positive association in medium- to low-income countries [9].

Overweight and obesity can have consequences not only for physical health, but also for mental health and quality of life in children and adolescents. Several studies indicated lower health-related quality of life (HRQoL) in children and adolescents with obesity [10, 11]. Many studies showed reduced values in the physical and social dimensions of HRQoL in childhood and adolescent overweight and obesity [10, 12–14]. Regarding associations between overweight or obesity and the psychological well-being, parent-, and school-related dimensions of HRQoL, previous findings were mixed [10–16]. Some former studies indicated that the extent of the overweight/obesity-related HRQoL impairment is higher in girls than in boys [13, 14] and in younger than in older adolescents [14].

Studies on somatoform complaints in children and adolescents with overweight and obesity are sparse, especially in younger children. A German study suggested a higher rate of somatoform disorders in adolescents with extreme obesity [17]. Children and adolescents with overweight also showed more sleep problems [18, 19] and suffered more frequently from headaches, back pain, and functional gastrointestinal disorders [20–22].

Regarding behavioral difficulties, externalizing as well as internalizing problems have been described more frequently in children and adolescents with overweight [3]. Many studies reported more peer-relationship problems in children and adolescents with overweight and obesity compared to those with normal weight [23, 24]. Concerning associations with hyperactive/inattentive behavior or emotional symptoms, previous findings were inconsistent [17, 23–27]. In terms of prosocial behavior and conduct problems, most studies showed no association with overweight and obesity in children and adolescents [23, 24].

The studies presented above highlight a potential link between overweight or obesity and mental health problems of children and adolescents. However, most previous studies included only a small number of participants with obesity in a limited age range and assessed only a few mental health issues. Studies on associations between overweight and somatoform complaints are especially rare.

Furthermore, while some studies suggested differences in strengths of associations between different age groups or sexes, the exact role of age and sex as well as socioeconomic parameters remains unclear. Therefore, this study aimed to explore the relationship between BMI-SDS (body mass index Standard Deviation Score) and SES, HRQoL, somatoform complaints, and behavioral difficulties in a large study population, with a specific focus on differences in the strengths of associations depending on age, sex, or SES. Based on previous studies, we hypothesized that BMI-SDS is positively associated with somatoform complaints and behavioral difficulties, and negatively associated with SES and HRQoL. In addition, we expected the associations between BMI-SDS and the psychological variables to be stronger in girls, older children/adolescents, and children and adolescents from families with lower SES.

## Methods

### Participants

The data for the study were collected between 2011 and 2020 within the LIFE Child study conducted in Leipzig. The LIFE Child study is a cohort study examining the healthy development of children and adolescents and the development of civilization diseases. Participants are examined every year from the prenatal period to young adulthood. From the age of 6 years, LIFE Child recruits children with obesity in a specific cohort (obesity cohort), leading to a higher proportion of obesity from this age on. The children and adolescents complete an elaborate study program including the collection of bio samples, body function tests, the assessment of personal characteristics, and many other examinations. For this purpose, a broad-based team of physicians, psychologists, nutritionists, and sports scientists is involved [28, 29]. The study was designed according to the criteria of the Declaration of Helsinki and approved by the Ethics Committee of the Medical Faculty of the University of Leipzig (Reg. No. 264/10-ek). All families participate voluntarily, and parents provide written informed consent before the inclusion of their children. Additionally, adolescents aged 12 years and older provide their own written informed consent.

In the present project, children and adolescents aged 4 to 18 years were assessed. The original dataset comprised 11631 data points. Data of children and adolescents for whom information on BMI (body mass index), sex, age, or socioeconomic status (SES) was missing were excluded (*n*=785). In the case of multiple visits of the same participant, we excluded all but the last study visits (*n*=7566). Furthermore, we restricted our analysis to the youngest child in the family, i.e., we excluded older siblings (*n*=930). This strategy prevented biological dependencies and led to a homogeneous age distribution within the sample. The final sample comprised 2350 children and adolescents (1150 girls and 1200 boys, mean age 10.87 years, age range 4.00 - <19). Based on child/adolescent age and the person who completed the questionnaires, we divided the total sample into two subsamples, a child sample (aged 4 to 10 years, questionnaires completed by parents, *n*=1213 (591 girls), mean age 7.32 years (SD=2.06)) and an adolescent sample (aged 11 to 18 years, questionnaires completed by adolescents themselves, *n*=1137 (559 girls), mean age 14.66 (SD=2.17)). However, the specific analyses were only performed in those participants who had completed the respective questionnaires.

### Assessments

#### Overweight/ obesity and BMI-SDS

BMI (kg/m^2^) was calculated from objectively measured height (without shoes) and weight (in underwear) collected by certified study assistants and transformed to BMI Standard Deviation Scores (BMI-SDS) according to German gender- and age-specific reference data [30]. We categorized participants into four weight groups. Children and adolescents with a BMI-SDS ≥ -1.282 and ≤ 1.282 (≥10th percentile, ≤ 90th percentile) were classified as "normal weight"; those with a BMI-SDS < -1.282 (<10th percentile) as “underweight”; those with a BMI-SDS > 1.282 and ≤ 1.881 (> 90th percentile, ≤ 97th percentile) as "overweight"; and those with a BMI-SDS > 1.881 (> 97th percentile) as "obese" [31].

#### Socio-economic status

For the assessment of SES, parents provided information on their education, their occupation, and their household net income [32]. The information was combined into a SES composite score ranging from 3 to 21 (adapted to the Winkler Index [32]), with higher scores indicating higher SES [32]. Based on cut-offs gained in a large representative German sample, this score can be used to categorize the SES into low (3.0 to 8.4 points), middle (8.5 to 15.4 points), and high (15.5 to 21.0 points) [32]. In a representative sample, the distribution of SES is expected to be 20% low, 60% middle, and 20% high [32].

#### Health-related quality of life (KIDSCREEN-27)

To evaluate HRQoL, we applied the self-report version of the questionnaire KIDSCREEN-27 [33]. This questionnaire was only applied in the adolescent sample, not in the child sample. The questionnaire consists of 27 questions answered on a 5-point Likert scale [33]. In total, 5 different dimensions were surveyed: physical well-being (5 items), psychological well-being (7 items), relationship with parents and personal autonomy (7 items), social support and peer group integration (4 items), and school environment (4 items). The scores of the individual questions were combined to sum scores representing the single dimensions. These scores were transformed to t-values (mean=50, SD=10) based on gender- and age-specific references. Higher values indicated higher HRQoL [33].

#### Somatoform complaints

Somatoform symptoms were assessed using a short version of the Giessen Complaints Questionnaire for Children and Adolescents (GBB-KJ) [34]. The single items assess headache, stomach ache, back pain, depressed mood, irritability, nervousness, sleep problems, and dizziness. The items capture the frequency of symptoms on an ordinal scale, ranging from "never/rarely", "once per month", "nearly every week", "several times per week", to "nearly every day" (corresponding to scores from 0 to 4). For the present analyses, all item responses were summed up to a sum score ranging from 0 to 32, with higher scores indicating more frequent somatoform symptoms.

#### Behavioral strengths and difficulties

Behavioral strengths and difficulties were assessed using the Strengths and Difficulties Questionnaire (SDQ) [35, 36]. It comprises 25 questions rated on an ordinal scale. Response categories are "not true", "somewhat true", and "certainly true." The item scores were combined into 5 sum scores: prosocial behavior, hyperactivity/ inattention, emotional symptoms, conduct problems, and peer relationship problems. Each score ranged from 0 to 10, with higher scores indicating more behavioral strengths (in the case of prosocial behavior) or difficulties (in all other cases).

#### Statistical analysis

The statistical analyses and visualization were performed using R 4.1.1. Continuous data were described in terms of means and standard deviations (SD), and categorical variables were described in terms of counts and percentages. Differences in score means between participants with normal or underweight and participants with overweight or obesity were assessed using two-tailed t-test.

To assess the associations between BMI-SDS and SES, we applied linear regression analysis. SES (categorical) was included as independent variable, and BMI-SDS (continuous) was included as the dependent variable. The association was adjusted for sex and age.

The associations between BMI-SDS (as independent variable) and HRQoL, somatoform symptoms, and behavioral strengths and difficulties (as dependent variables) were examined using linear regression analyses. All associations were adjusted for sex, age, and SES.

To assess whether or not the strengths of associations between BMI-SDS and HRQoL, somatoform symptoms, and behavioral strengths and difficulties differed depending on the covariates, the above-mentioned associations were checked for interactions between BMI-SDS and age, sex, and SES. For interaction with age, we divided the child sample into kindergarten age (from 4.00 to 6.49 years) and primary school age (from 6.50 to 10.99 years). Similarly, we split the adolescent sample into early puberty age (from 11.00 to 13.99 years) and late puberty age (from 14.00 to 18.99 years). The interactions were only considered if the model quality was retained, i.e., if the interaction term did not induce strong variance inflation (variance inflation factor<5).

Effects were reported as non-standardized regression coefficients (beta). Associations and interactions with a *p*-value<0.05 were considered significant.

## Results

### Descriptive analysis

The total sample included 2350 participants (1150 (48.9%) girls, mean age=10.87 years, SD=4.24) (Table 1). On average, participants had a BMI-SDS of 0.34 (SD=1.25). Most children and adolescents were categorized as "normal weight" (*n*=1660, 70.6%), whereas 352 (15.0%) were categorized as “obese”, 169 (7.2%) as “overweight”, and 169 (7.2%) as “underweight”. Regarding SES, most children and adolescents (*n*=1335, 56.8%) had middle SES, while 730 (31.1%) had high SES, and 285 (12.1%) had low SES. Table 2 shows the average scores of the single scales of the questionnaires stratified by weight status. With a few exceptions (quality of life regarding school environment, somatoform complaints, prosocial behavior, and hyperactivity/inattention (only in adolescent sample)), all differences between children/adolescents with normal or underweight and children/adolescents with overweight or obesity were statistically significant (*p* < .05).

**Table 1:** Characteristics of the study samples

	**Study sample** (*n*=2350)		**Child sample** (age 4 to 10, *n*=1213)		**Adolescent sample** (age 11 to 18, *n*=1137)	
	M (SD)	n (%)	M (SD)	n (%)	M (SD)	n (%)
**Sex**						
Female		1150 (48.9%)		591 (48.7%)		559 (49.2%)
Male		1200 (51.1%)		622 (51.3%)		578 (50.8%)
**Age**	10.87 (4.24)		7.32 (2.06)		14.66 (2.17)	
**SES**^a^						
Low		285 (12.1%)		127 (10.5%)		158 (13.9%)
Middle		1335 (56.8%)		658 (54.2%)		677 (59.5%)
High		730 (31.1%)		428 (35.3%)		302 (26.6%)
**BMI-SDS**^b^	0.34 (1.25)		0.18 (1.15)		0.51 (1.33)	
**Weight status**^c^						
Underweight		169 (7.2%)		80 (6.6%)		89 (7.8%)
Normal weight		1660 (70.6%)		930 (76.7%)		730 (64.2%)
Overweight		169 (7.2%)		68 (5.6%)		101 (8.9%)
Obesity		352 (15.0%)		135 (11.1%)		217 (19.1%)

^a^SES: Socioeconomic status

^b^BMI-SDS: BMI Standard Deviation Score (mean=0, SD=1)

^c^underweight: BMI-SDS<-1.282, normal weight: -1.282≤BMI-SDS≤1.28, overweight: 1.282<BMI-SDS≤1.881, obese: BMI-SDS>1.881

**Table 2:** Health-related quality of life, somatoform complaints, and behavioral strengths and difficulties in the present sample

	**Total sample**	**Children with normal weight/ underweight**	**Children with overweight/ obesity**	**Difference underweight/ normal weight and overweight/ obesity**
**Health-related quality of life** (**KIDSCREEN-27**, *n*=1118, age: 11-18 years)				
Physical well-being	49.03 (9.40)	50.59 (9.39)	44.89 (8.08)	***
Psychological well-being	49.38 (10.00)	50.01 (10.05)	47.75 (9.70)	***
Autonomy & parents	53.66 (10.01)	54.12 (9.78)	52.45 (10.52)	*
Peers & social support	51.78 (10.57)	52.23 (9.82)	50.60 (12.27)	*
School environment	50.95 (9.49)	51.11 (9.50)	50.53 (9.49)	
**Somatoform complaints** (**GBB-KJ**^a^ **parent reports**, *n*=762, age: 4-10 years)				
Sum score	3.65 (3.70)	3.56 (3.52)	4.32 (4.71)	
**Somatoform complaints** (**GBB-KJ**^a^ **self-report**, *n*=542, age: 11-18 years)				
Sum score	6.48 (6.19)	6.39 (6.26)	6.78 (5.96)	
**Behavioral strengths and difficulties** (**SDQ**^b^ **parent reports**, *n*=1186, age: 4-10 years)				
Prosocial behavior	7.91 (1.69)	7.91 (1.69)	7.89 (1.68)	
Hyperactivity/inattention	3.80 (2.43)	3.74 (2.43)	4.10 (2.40)	
Emotional symptoms	1.89 (1.89)	1.80 (1.85)	2.36 (2.04)	***
Conduct problems	2.12 (1.63)	2.05 (1.56)	2.51 (1.92)	**
Peer relationship problems	1.28 (1.57)	1.11 (1.45)	2.13 (1.86)	***
**Behavioral strengths and difficulties** (**SDQ**^b^ **self-report**, *n*=1042, age: 11-18 years)				
Prosocial behavior	7.82 (1.84)	7.88 (1.80)	7.67 (1.95)	
Hyperactivity/inattention	3.54 (2.22)	3.46 (2.26)	3.77 (2.12)	*
Emotional symptoms	2.56 (2.18)	2.47 (2.17)	2.79 (2.19)	*
Conduct problems	1.70 (1.45)	1.55 (1.37)	2.07 (1.58)	***
Peer relationship problems	2.30 (1.77)	2.05 (1.63)	2.94 (1.95)	***

^a^short version of the Giessen Complaints Questionnaire for Children and Adolescents

^b^Strengths and Difficulties Questionnaire

^***^p<0.001, **p<0.01, *p<0.05

### Association between BMI-SDS and SES

In the total sample, children and adolescents with low SES had a significantly higher BMI-SDS than those with high SES (b=0.95, *p*<0.001), or those with middle SES (b=0.65, *p*<0.001). In children with a high SES, the average BMI-SDS was estimated 0.05 (SD=1.04), compared to 0.35 (SD=1.25) in those with middle SES, and 1.00 (SD=1.48) in those with a low SES.

## Associations between BMI-SDS and Health-related quality of life (HRQoL)

The results of the analyses are presented in more detail in Table 3. The analyses of the adolescent sample revealed a significant association between higher BMI-SDS and lower scores on the physical well-being scale of the KIDSCREEN-27 (beta=-1.56, *p*<0.001). A significant interaction with age showed that this association was weaker at late (beta=-0.84, *p*<0.001) than at early puberty age (beta=-2.67, *p*=0.001).

**Table 3:** Associations between BMI-SDS and health-related quality of life, somatoform complaints, and behavioral difficulties in the adolescent sample (11 to 18 years)

	**BMI-SDS**			
	**beta**	**95% CI**	**p**	**significant interactions (beta)**
**Health-related quality of life (KIDSCREEN-27)**				
** Physical well-being**	**-1.56**	-1.95 to -1.16	<0.001	with age^c^ (1.83***)
** Psychological well-being**	**-0.87**	-1.29 to -0.44	<0.001	with age^c^ (1.62***)
** Autonomy & parents**	-0.25	-0.69 to 0.19	0.27	with age^c^ (1.26**)
** Peers & social support**	-0.38	-0.86 to 0.09	0.12	with age^c^(1.74***)
** School environment**	-0.03	-0.46 to 0.39	0.87	with age^c^ (1.19**)
**Somatoform complaints (GBB-KJ**^a^)				
** Sum score**	0.19	-0.21 to 0.59	0.35	
**Behavioral strengths and difficulties (SDQ**^b^)				
** Prosocial behavior**	-0.03	-0.12 to 0.05	0.46	
** Hyperactivity/Inattention**	0.03	-0.08 to 0.13	0.63	
** Emotional symptoms**	**0.10**	0.00 to 0.19	0.04	with age^c^ (-0.25**)
** Conduct problems**	**0.14**	0.08 to 0.21	<0.001	with age^c^ (-0.18**)
** Peer relationship problems**	**0.27**	0.19 to 0.35	<0.001	

All associations were adjusted for sex, age, and SES (Socioeconomic status)

BMI-SDS: adjusted for sex and age

beta: non-standardized regression coefficient

*CI:* Confidence interval

^a^short version of the Giessen Complaints Questionnaire for Children and Adolescents

^b^Strengths and Difficulties Questionnaire

^c^Reference: early puberty age

^***^p<0.001, **p<0.01, *p<0.05

Furthermore, a significant negative association between BMI-SDS and scores on the psychological well-being scale was shown (beta=-0.87, *p*<0.001). Again, a significant interaction with age suggested that the relationship was weaker at late (beta=-0.22, *p*=0.42) than early puberty age (beta=-1.84, *p*<0.001).

Although there was no significant association between BMI-SDS and the KIDSCREEN scales "autonomy and parents (ap)", “peers and social support (ps)” as well as “school environment (se)”, significant interactions with age (p_ap_=0.005, p_ps_<0.001 and p_se_=0.006) showed that the negative associations at early puberty age (beta_ap_=-1.02 (*p*=0.004), beta_ps_=-1.44 (*p*<0.001), and beta_se_=-0.75 (*p*=0.03)) turned into marginally positive associations at late puberty age (beta_ap_=0.25 (*p*=0.39, beta_ps_=0.30 (*p*=0.33) and beta_se_=0.44 (*p*=0.11)). The analyses revealed no significant moderation of the associations by sex or SES.

### Associations between BMI-SDS and somatoform complaints

We observed no significant association between somatoform complaints in GBB-KJ and BMI-SDS, neither in the child sample (beta=0.20, *p*=0.14, see table 4), nor in the adolescent sample (beta=0.19, *p*=0.35, see table 3). However, in the child sample, a significant interaction with age (*p*=0.002) indicated that the association changed from negative at kindergarten age (beta=-0.41, *p*=0.09) into positive at primary school age (beta=0.47, *p*=0.003). We did not find significant interactions with sex or SES.

**Table 4:** Associations between BMI-SDS and somatoform complaints and behavioral difficulties in the child sample (4 to 10 years)

	**BMI-SDS**			
	**beta**	**95% CI**	**p**	**significant interactions (beta)**
**Somatoform complaints (GBB-KJ**^a^)				
**Sum score**	0.20	-0.06 to 0.46	0.14	with age^c^ (0.88**)
**Behavioral strengths and difficulties (SDQ**^b^)				
**Prosocial behavior**	0.02	-0.07 to 0.10	0.70	with age^c^ (0.25**)
**Hyperactivity/Inattention**	0.04	-0.08 to 0.17	0.47	with SES^d^ (0.38*)
**Emotional symptoms**	0.07	-0.02 to 0.17	0.14	with age^c^ (0.30**) with SES^d^ (0.30*)
**Conduct problems**	**0.15**	0.07 to 0.24	<0.001	
**Peer relationship problems**	**0.24**	0.16 to 0.32	<0.001	with age^c^ (0.34***)

All associations were adjusted for sex, age, and SES (Socioeconomic status)

BMI-SDS: adjusted for sex and age

beta: non-standardized regression coefficient

*CI:* Confidence interval

^a^short version of the Giessen Complaints Questionnaire for Children and Adolescents

^b^Strengths and Difficulties Questionnaire

^c^Reference: kindergarten age

^d^Reference: low SES

^***^p<0.001, **p<0.01, *p<0.05

### Associations between BMI-SDS and behavioral strengths and difficulties

Table 3 (adolescent sample) and Table 4 (child sample) show the coefficients for the associations between the BMI-SDS and the different SDQ scales.

**Fig. 1:**
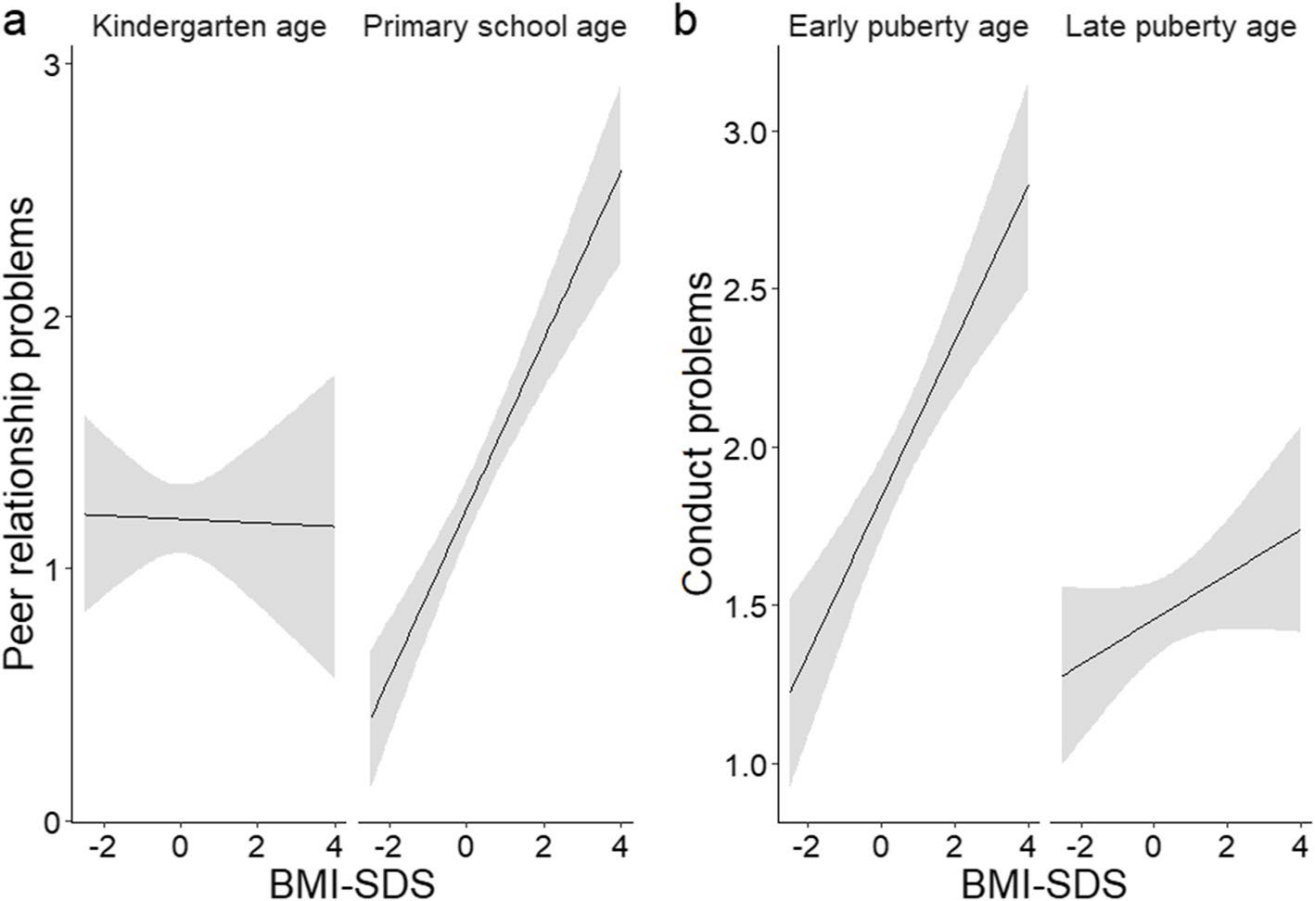
Interaction of age with associations between BMI-SDS and behavioral difficulties (panel a: Peer relationship problems in 1186 children between 4 and 10 years, panel b: Conduct problems in 1042 children and adolescents between 11 and 18 years)

In the child sample (4–10 years), there was a significant positive association between BMI-SDS and parent-reported conduct problems (beta=0.15, *p*<0.001). Moreover, we found a significant positive association between BMI-SDS and parent-reported peer relationship problems (beta=0.24, *p*<0.001). Here, a significant interaction with age (*p*<0.001, see Figure 1) indicated that the positive relation existed only at primary school age (beta=0.33, *p*<0.001), not at kindergarten age (beta=-0.01, *p*=0.92). The scores on the SDQ scales “prosocial behavior”, “hyperactivity/inattention”, and “emotional symptoms” were not significantly associated with BMI-SDS in the child sample. However, regarding the association between BMI-SDS and prosocial behavior (ps) and emotional symptoms (es), respectively, significant interactions with age (p_ps_=0.009 and p_es_=0.006) revealed a change from negative associations at kindergarten age (beta_ps_=-0.16 (*p*=0.049) and beta_es_=-0.14 (0.08) into positive associations at primary school age (beta_ps_=0.09 (*p*=0.08) and beta_es_ =0.15 (*p*=0.007). Furthermore, the associations between BMI-SDS and hyperactivity/inattention (hi) and emotional symptoms, respectively, revealed significant interactions with SES (p_hi_=0.02 and p_es_=0.02), showing changes in the associations from negative at low SES (beta_hi_=-0.24 (*p*=0.10) and beta_es_=-0.14 (*p*=0.21) into positive at middle SES (beta_hi_=0.14 (*p*=0.08) and beta_es_=0.16, (*p*=0.01)). The analyses revealed no interactions with sex.

In the adolescent sample, we found a significant positive association between BMI-SDS and scores on the SDQ scale emotional symptoms (beta=0.10, *p*=0.04). A significant interaction with age (*p*=0.006) revealed that the positive relation at early pubertal age (beta=0.24, *p*<0.001) was no longer present at late pubertal age (beta=-0.01, *p*=0.85). As in the child sample, we observed a significant positive association between BMI-SDS and the score on the conduct problems scale (beta=0.14, *p*<0.001). A significant interaction with age (*p*=0.007, see Figure 1) showed that this association was weaker at late (beta=0.07, *p*=0.11) than at early puberty age (beta=0.25, *p*<0.001). Comparable with the child sample, there was a significant positive association between BMI-SDS and scores on the peer relationship problems scale (beta=0.27, *p*<0.001). Sex and SES were not found to moderate the associations between BMI-SDS and behavioral difficulties in the adolescent sample.

## Discussion

This study investigated associations between a wide range of mental health parameters and BMI-SDS in a large sample of German children and adolescents aged 4 to 18 years. In addition, it assessed whether these associations were moderated by sex, age and SES. We were able to show that a higher BMI-SDS was positively associated with self- or parent-reported behavioral difficulties, inversely associated with SES and HRQoL, and not associated with prosocial behavior. Most interestingly, we observed no significant association between BMI-SDS and somatoform complaints, stronger associations between BMI-SDS and mental health in primary school age and early puberty age (compared to younger or older children or adolescents), and an influence of SES on some associations between BMI-SDS and behavioral difficulties in children. The study is clinically significant because it highlights the mental health limitations in children and adolescents with higher BMI-SDS and thus the importance of including psychosocial interventions in overweight/obesity treatment.

### Study sample and association between BMI-SDS and SES (total sample)

In the present sample, 7.2% of children were overweight (but not obese), and 15.0% were obese. The large proportion of children and adolescents with obesity can be explained by the fact that there is an obesity cohort in the LIFE Child study for which children and adolescents with obesity are explicitly recruited [28].

As hypothesized, the analyses revealed a negative association between BMI-SDS and SES, consistent with results of previous studies conducted in high-income countries [6, 9]. A study by Gibbs et al. identified unhealthier feeding practices as the most important explanation for the association between SES and early childhood obesity [37]. Healthy foods are often more expensive and therefore less likely to be purchased by families with low SES/low income [38].

### Association between BMI-SDS and HRQoL (adolescent sample)

Our analyses showed lower HRQoL in adolescents with higher BMI-SDS. However, only the associations between BMI-SDS and physical and psychological well-being were significant. Regarding the other domains of quality of life, the associations with BMI-SDS pointed in the expected direction but did not reach statistical significance.

The finding that physical well-being was lower in adolescents with higher BMI-SDS is in line with previous findings [12, 13, 15]. An explanation might be reduced physical activity and fitness in adolescents with higher BMI [39]. A study by Mozzillo et al. was able to show that lower HRQoL was associated with less physical activity among adolescents with overweight or obesity [40].

Regarding psychological well-being, previous studies showed lower levels in children and adolescents with obesity compared to children and adolescents with normal weight [10, 15] but no differences between adolescents with overweight (including obesity) and normal weight [13]. The present finding, however, suggests a linear relationship between BMI-SDS and psychological well-being. One explanation is that adolescents with higher BMI suffer more frequently from social withdrawal, social isolation or bullying [41–43]. Furthermore, adolescents with higher BMI might be exposed to stigmatization by parents and teachers [44, 45]. These factors are likely to have a negative effect on the psychological well-being of the affected person.

In line with another study that found greater HRQoL reductions of higher weight status relative to normal weight in younger than in older adolescents [14], associations between BMI-SDS and physical as well as psychological well-being were stronger at early than in later puberty age. Regarding autonomy and parents, peers and social support, and school environment, we observed the same trend. These findings indicate that associations between BMI-SDS and HRQoL are particularly strong in young adolescents, i.e., during early puberty. In that specific age group, adolescents might be particularly sensitive to appearances and teasing, e.g., due to limited social functioning. In line, Riazi et al. showed lower social functioning in pre-pubescents with obesity than in pubescents and post-pubescents with obesity [46].

In contrast to age, sex or SES did not moderate associations between BMI-SDS and HRQoL. Some previous studies suggest that the overweight/obesity-related HRQoL impairment is higher in girls than in boys [13, 14]. The fact that we did not find a difference could be due to the fact that our sample was smaller compared to the two studies mentioned above.

### Association between BMI-SDS and somatoform complaints (child and adolescent sample)

While previous studies pointed to a significant association between BMI and somatoform complaints [17, 18, 20–22, 47], here, associations pointed in the expected direction but did not reach significance. One reason could be the smaller sample size (compared to the samples on associations with HRQoL and behavioral strengths and difficulties). Furthermore, our sample included healthy children and adolescents that rarely reported somatoform complaints. Interestingly, in the child sample, a significant interaction revealed that the association between BMI-SDS and somatoform complaints becomes apparent only from the primary school age onwards. Similar to the association between BMI-SDS and HRQoL, associations between BMI-SDS and somatoform complaints might be more relevant in late pre-puberty/early puberty.

### Association between BMI-SDS and behavioral strengths and difficulties (child and adolescent sample)

As expected, children and adolescents with higher BMI-SDS showed more behavioral difficulties than those with lower BMI-SDS, especially more internalizing problems (emotional symptoms (the latter only in the adolescent sample) and peer relationship problems) and more conduct problems.

As already highlighted above, the associations between higher BMI-SDS and internalizing problems might be explained by more social withdrawal, social isolation, bullying, and exposed stigmatization in children and adolescents with higher BMI-SDS [41–45].

The finding of significantly more conduct problems in children and adolescents with higher BMI-SDS is more surprising, as previous studies did not find such association [23, 24]. Various studies have documented an altered hormone balance in children and adolescents with obesity [48, 49]. Since various hormones can also influence behavior, this could be a reason for more frequent conduct problems in children and adolescents with higher BMI-SDS. As already shown for HRQoL and somatoform complaints, significant interactions with age indicate that associations between BMI-SDS and internalizing and conduct problems are more serious in late prepuberty or early puberty than in earlier childhood or later adolescence.

Regarding hyperactivity and inattention, we did not find a significant association with BMI-SDS. This is in line with previous studies using the same instrument (SDQ) [23, 24]. In contrast, several studies assessing clinically relevant amounts of hyperactivity/inattention (e.g., diagnosis of ADHS) showed positive associations with higher BMI [24, 25, 27]. Therefore, it is possible that associations between BMI and symptoms of hyperactivity/inattention are only observable in a range that is not captured by the SDQ.

In the child sample, we found a significant interaction with SES for the associations between BMI-SDS and hyperactivity/inattention and emotional problems. The associations for low SES pointed in a negative direction and for middle SES in a positive direction, whereas the trend for high SES was in between. We have no explanation for this inconclusive result, since we expected a falling or rising trend from low to middle to high SES.

### Strengths and limitations

One strength of this study was the examination of many different psychosocial factors in a large German sample of children and adolescents. A special characteristic was the consideration of interactions with sex, age, and SES.

As low SES was underrepresented in our sample, the findings can be transferred to the general population only to a limited extent [32]. Two further limitations concern the collection of data via questionnaires. First, responses might be biased, e.g., by social desirability. Second, responses provided by parents and children themselves might differ and, therefore, results of both the child sample and the adolescent sample are not necessarily comparable.

## Conclusion

A higher BMI-SDS in children and adolescents is associated with more behavioral difficulties and lower HRQoL. The findings suggest that some associations are especially strong in the pre-pubertal or early pubertal stage of development or at middle SES, while gender does not moderate the strengths of associations. The findings highlight the importance of mental problems in children and adolescents with higher BMI-SDS and, consequently, the relevance of including psychological interventions in the treatment of overweight and obesity.

## Data Availability

The datasets generated and/or analyzed during the current study are not publicly available due to ethical restrictions. The LIFE Child study is a study collecting potentially sensitive information. Publishing data sets is not covered by the informed consent provided by the study participants. Furthermore, the data protection concept of LIFE requests that all (external as well as internal) researchers interested in accessing data sign a project agreement. Researchers that are interested in accessing and analyzing data collected in the LIFE Child study may contact the data use and access committee (forschungsdaten@medizin.uni-leipzig.de). Furthermore, we thank Youth in Mind very much for permission to use the SDQ (Strengths and Difficulties Questionnaire).

## Abbreviations

Beta: non-standardized regression coefficients
BMI: body mass index
BMI-SDS: body mass index Standard Deviation Score
GBB-KJ: short version of the Giessen Complaints Questionnaire for Children and Adolescents
HRQoL: health-related quality of life
KiGGS: German Health Interview and Examination Survey for Children and Adolescents
SES: socioeconomic status
SD: standard deviation
SDQ: Strengths and Difficulties Questionnaire
